# Effectiveness of Pharmacy Automation Systems Versus Traditional Systems in Hospital Settings: A Systematic Review

**DOI:** 10.7759/cureus.77934

**Published:** 2025-01-24

**Authors:** Enaam M Shbaily, Ibrahim M Dighriri, Norah S Alotaibi, Razan M Alqahtani, Ali M Mushawwal, Abdulrahman G Mohammed, Ghada S Barwaished, Maher M Almalki, Milaf Alshammari, Shahad B Alharbi, Saad M Almalki, Hanaa A Alatawi, Shamael A Alsharif, Mohammed Almurayt

**Affiliations:** 1 Department of Pharmacy, Armed Forces Hospital Jazan, Jazan, SAU; 2 Department of Pharmacy, King Abdulaziz Specialist Hospital, Taif, SAU; 3 College of Pharmacy, Taif University, Taif, SAU; 4 College of Pharmacy, Princess Nourah bint Abdulrahman University, Riyadh, SAU; 5 Department of Pharmacy, Al Nahdi Medical Company, Jazan, SAU; 6 Department of Pharmacy, General Directorate for Prison Health in Medical Service-Ministry of Interior (MOI), Jazan, SAU; 7 College of Pharmacy, King Saud University, Riyadh, SAU; 8 Department of Pharmacy, Al Nahdi Medical Company, Makkah, SAU; 9 College of Pharmacy, University of Hafr Albatin, Hafr Albatin, SAU; 10 College of Pharmacy, Qassim University, Qassim, SAU; 11 Department of Pharmacy, University of Tabuk, Tabuk, SAU; 12 College of Pharmacy, Umm Al-Qura University, Makkah, SAU; 13 Department of Pharmacy, Armed Forces Hospital, Abha, SAU

**Keywords:** hospital pharmacy, medication errors, operational efficiency, patient safety, pharmacy automation, systematic review

## Abstract

Medication errors (MEs) in hospital settings remain a significant global healthcare challenge, resulting in adverse patient outcomes, increased healthcare costs, and reduced operational efficiency. Traditional pharmacy systems (TPS) are particularly vulnerable to human error, inefficient inventory management, and workflow bottlenecks. While pharmacy automation systems (PAS) have emerged as a potential solution, there is a notable gap in the literature regarding comprehensive comparative studies between PAS and TPS across multiple outcomes and settings. This systematic review addresses this gap by evaluating the comparative effectiveness of PAS versus TPS in hospital settings, focusing on MEs, operational efficiency, cost-effectiveness, and patient outcomes.

We conducted a systematic literature review following the Preferred Reporting Items for Systematic Reviews and Meta-Analyses (PRISMA) guidelines, searching PubMed and Cochrane Library databases for studies published between 2010 and June 2024 that directly compared PAS with TPS in hospital settings. The review examined various PAS technologies, including centralized pharmacy robots, automated dispensing cabinets (ADCs), and hybrid systems incorporating centralized and decentralized technologies. Of 1,085 studies initially identified, 32 met the inclusion criteria for comprehensive analysis. The overall mean effect size was 0.505 (95% confidence interval (CI): 0.487 to 0.523), indicating a moderately positive effect of PAS implementation. Key findings demonstrated that PAS significantly reduced MEs, particularly in automated dispensing systems (ADS) and computerized physician order entry (CPOE) systems. While initial implementation costs were substantial, long-term operational costs were significantly lower due to reduced labor requirements and medication wastage. Workflow efficiency improvements enabled pharmacists to dedicate more time to clinical activities. Patient outcomes improved through enhanced medication safety and reduced adverse drug events. This review provides robust evidence supporting PAS implementation in hospitals. It demonstrates that despite significant initial investment requirements, the long-term benefits in error reduction, operational efficiency, and patient safety justify implementation. Future research should focus on detailed cost-benefit analyses across various hospital settings and assessments of staff satisfaction to optimize implementation strategies.

## Introduction and background

Pharmacy automation systems (PAS) have recently emerged as a potential solution for relieving the strain of managing medications in hospital settings [1]. These systems are designed to improve patient safety and efficiency and reduce costs related to traditional manual dispensing methods. Traditional pharmacy systems (TPS) have been associated with significant medication errors (MEs), with studies indicating 2%-5% error rates in medication dispensing and administration, resulting in adverse patient outcomes and estimated annual costs of $2-$5 billion globally [1-3]. These errors stem from manual processing limitations, inventory mismanagement, and workflow inefficiencies that impact patient safety and operational productivity. While various PAS technologies have emerged, including centralized robotics for medication storage and dispensing, automated dispensing cabinets (ADCs) for point-of-care access, computerized physician order entry (CPOE) systems, automated compounding systems, and barcode medication administration systems, there remains a notable gap in the literature regarding their comparative effectiveness against traditional systems [1-3]. Existing studies have typically focused on individual aspects of automation or specific settings, lacking comprehensive analyses that evaluate multiple outcomes across different hospital environments. This systematic review addresses this gap by providing a thorough comparative analysis of PAS versus TPS across four critical domains: medication error rates, operational efficiency, cost-effectiveness, and patient outcomes, offering healthcare decision-makers robust evidence for evaluating automation implementation strategies [1-4].

The rapid demand for hospital-based healthcare services, coupled with increasingly complex courses of medication and treatment, has placed significant pressure on hospitals to ensure safe, accurate, and efficient delivery of drugs [1,2]. Traditional manual systems are highly susceptible to human error when dispensing medications. Moreover, they are slow and cumbersome, particularly regarding inventory management. Many hospitals have installed automated drug distribution systems (ADS) to address this challenge [2]. The PAS includes centralized pharmacy robots, decentralized ADC, and hybrid systems that incorporate aspects of both centralized and decentralized technologies. These systems are engineered to improve the efficiency of the medication use process, from prescribing to administering treatments, reducing the probability of errors in medication usage while optimizing resource utilization and workflow efficiency [3,4]. One or more steps in medication management may reduce errors and increase overall efficiency. The study states that the benefits and advantages of implementing integrated solutions in centralized pharmacies and hospital wards have not been comprehensively analyzed [1,4].

This systematic review assessed PAS's impact on manual systems traditionally used in hospitals. It will also synthesize evidence of substantial value to healthcare decision-makers contemplating pharmacy automation implementation or enhancement within their organizations.

## Review

Methods

The present systematic review was conducted per the Preferred Reporting Items for Systematic Reviews and Meta-Analyses (PRISMA) guidelines [5].

Search Strategy

The search strategy was intended to encompass all aspects of the research questions, including the impact and effectiveness of PAS in hospitals. The approach used a broad literature search to determine available studies comparing ADC, automated medication management systems, robotic pharmacy systems, and other automated systems with established manual systems in pharmacies. The search was performed to find reports on studies that examined outcomes, such as medication error rate, pharmacy efficiency, patient costs, and outcomes. PubMed, Cochrane Library, and Embase were the target databases for a comprehensive search for articles involving the maximum amount of comparable literature in the analysis. Keywords such as "pharmacy automation systems," "traditional pharmacy systems," "hospital settings," and "impact and effectiveness" and their synonyms were used to search articles across the databases.

Study Selection

The process was conducted systematically to ensure that all relevant studies were included and that the selection process was transparent and reproducible. Initial screening involved reviewing the titles and abstracts of all identified studies. In cases of uncertainty, if the information provided in the title and abstract was insufficient, all studies were considered for full-text review. After the initial screening, all eligible full-text articles were reviewed to ensure that they met predefined inclusion criteria.

Eligibility Criteria

Studies were screened and included based on the inclusion criteria: randomized controlled trials (RCTs), cohorts, case studies, and trials focusing on PAS in hospitals and the presence of a comparator group published from 2010 to June 2024. Additionally, studies were excluded based on the following exclusion criteria: absence of a comparator group, non-hospital setting, non-peer-reviewed articles, editorials, and opinion pieces.

Data Extraction

Data extraction was performed to extract relevant data from the included studies. A data extraction form was used to scrape the data from the studies to ensure that the extraction process remained comprehensive and consistent. Data such as the authors, study design, population, intervention, comparison, outcomes, and results were included. For each study, the reviewer documented key variables such as sample size, type of PAS assessed, type of TPS used for comparison, and specific effects measured and reported, including medication errors (MEs), efficiency, cost, and patient outcomes.

Risk of Bias Assessment

A detailed risk of bias assessment was performed for each study using the Risk of Bias in Non-randomized Studies of Interventions (ROBINS-I) tool. This tool assesses seven domains of bias: confounding, participant selection, intervention classification, deviations from intended interventions, missing data, outcome measurement, and selection of reported results. For each domain, studies were classified as having a low, moderate, severe, or critical risk of bias or no information. Each study's overall risk of bias was determined based on the highest risk level in any domain. A risk of bias summary table was created to present the assessment for each study across all domains. Additionally, a traffic light plot and a summary plot were generated to visually represent the distribution of the risk of bias across the included studies.

Results

**Figure 1:**
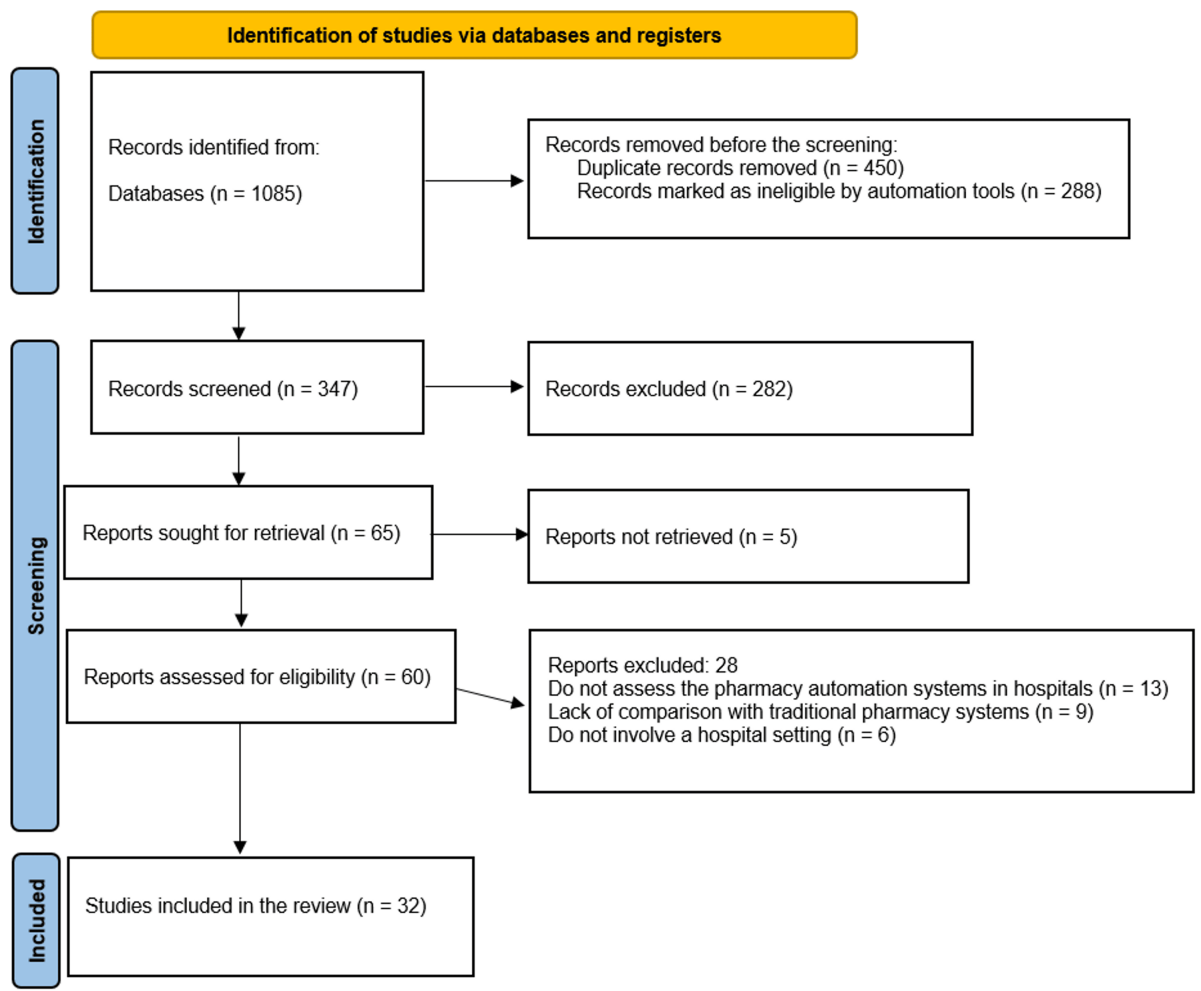
PRISMA flow diagram This figure illustrates the study selection process according to the PRISMA guidelines. PRISMA: Preferred Reporting Items for Systematic Reviews and Meta-Analyses

The initial search yielded 1,085 records identified through a database search. After deduplication, 450 records were removed, and 288 were excluded using automated tools as ineligible. This left 347 publications for the title and abstract screening. Of these, 282 records were excluded at this stage; therefore, the full texts of 65 potentially relevant papers were retrieved. Five of these papers could not be tracked, leaving 60 papers to assess their eligibility. Of these, 28 papers were excluded for three main reasons: 13 did not address the topic of PAS in hospitals, nine did not compare with TPS, and six were not set in hospitals. Finally, 32 studies met all criteria and were included in the final systematic review (Figure 1).

Technology Implementation in Medication Dispensing

Implementing technology in pharmaceutical distribution channels improves safety and efficiency in health-service settings. The study within the stated period indicated a significant reduction in errors due to the acceptance of computerized physician order entry (CPOE). As noted by Álvarez Díaz et al. (2010), this has been the case for a 1,070-bed hospital [6]. Zhang et al. (2022) reported that intelligent intravenous drug-dispensing robots significantly increased accuracy and efficiency, notably eliminating accidental hand stab injuries [7]. Although first-generation robotic systems are a barrier to implementation in practice, as documented by Nurgat et al. (2015), they reported intolerable failure rates regarding dose accuracy [8]. However, automated drug-dispensing technologies, when combined with pharmacist-led medication reviews, reduce drug-related problems (DRPs) in the elderly [9]. The overall body of evidence indicates that although technology implementation improves the accuracy and safety of medication dispensing, its successful integration requires staff training, technical support, and ongoing system monitoring (Table 1).

**Table 1:** Studies demonstrating the role of technology implementation in medication dispensing UDDS: unit dose dispensing system, CPOE: computerized physician order entry, ADS: automated dispensing system, IT: information technology, DRPs: drug-related problems, MEs: medication errors, FMEA: Failure Mode and Effects Analysis

Author	Study design	Population	Intervention	Comparison	Outcomes	Results
Álvarez Díaz et al. (2010) [6]	Prospective observational study	1,070-bed general hospital	Analyzed stock, UDDS (with/without CPOE), ADS (with/without CPOE)	Different dispensing systems	Error rates, stages, contributing factors	Significant reduction in errors with CPOE, highest errors in no-CPOE-ADS
Zhang et al. (2022) [7]	Observational study	Clinical nursing staff and patients	Intelligent intravenous drug-dispensing robot	Manual dispensing	Efficiency, residual amount, accidental hand stab injury, drug dispensing accuracy	Improved efficiency, reduced residual amount, no accidental injuries, higher accuracy compared to manual dispensing
Chen et al. (2013) [10]	Observational study	Medical center with 2,500 beds	Automatic robotic arm for dispensing chemotherapy	Manual chemotherapy dispensing	Accuracy, time efficiency, safety	Significant improvement in accuracy and time efficiency, enhanced safety
ElLithy et al. (2023) [11]	Descriptive mixed observational study	Tertiary hospital in the Gulf Cooperation Council, including pharmacy, medical personnel, and other staff	FMEA for pharmacy automation and robotics implementation	Manual workflow versus ADS	Identification of risks, root causes, and implementation of corrective measures	Revealed challenges such as staff training, technical issues, and inadequate communication; significant corrective steps taken to improve performance and safety
Escobar-Rodríguez et al. (2012) [12]	Case study	Spanish hospital with 200,000 covered individuals, 320 beds, 1,100 staff	Deployment of information technology and continuous control monitoring systems	Traditional manual processes	Prevention of MEs	Improved detection and correction of MEs, efficient process re-engineering
Schneider (2018) [13]	Observational study	US healthcare system	Technology implementation in pharmacy practice	Traditional pharmacy practices	Safety and efficiency in medicine use	Improved medication safety and effectiveness
Nurgat et al. (2015) [8]	Observational study	Saudi Arabian healthcare system	Implementation of a first-generation chemotherapy-compounding robot	Manual compounding processes	Compounding accuracy, system uptime, workflow disruptions	Limited efficiency impact, high failure rates with dose accuracy, technical and nontechnical challenges
Yaniv and Knoer (2013) [14]	Observational study	Cleveland Clinic, Taussig Cancer Center Pharmacy	Implementation of an IV-compounding robot for preparing chemotherapy doses	Manual compounding	Compounding accuracy, system uptime, and workflow disruptions	The robot prepared 7,384 medication doses; 85 (1.2%) exceeded the desired accuracy range (±4%)
Geersing et al. (2020) [15]	Comparative study	Centralized cytotoxic drugs preparation unit of the OLVG hospital, Amsterdam, Netherlands	Automated preparation using the APOTECAchemo robotic system	Manual compounding methods	Dose accuracy and precision	Robotic and manual compounding methods produced accurate cytostatic products within a 10% deviation of the prescribed amount
Kwint et al. (2011) [9]	Pragmatic randomized controlled study	Patients recruited from six Dutch community pharmacies	Pharmacist-led medication review of DRPs for patients using automated drug-dispensing systems	Regular reviewing	Reduction in the number of DRPs leading to a recommendation for drug change	Medication review significantly reduces DRPs in elderly patients using automated drug-dispensing systems

Economic Evaluation and Cost-Effectiveness

Economic evaluations and cost-effectiveness studies concerning PAS from 2015 to 2022 revealed positive monetary results across several healthcare settings. Baan et al. (2022) [16] and Liu et al. (2022) [17] proved that significant cost savings are accrued by vial-sharing through automated compounding systems specifically focused on high-cost medications. A financial study in the surgical intensive care unit (ICU) by Chapuis et al. (2015) showed a positive net present value due to reduced nurse time, drug storage costs, and expired medication waste [18]. The workforce impact study of ADC by Noparatayaporn et al. (2017) indicated that the requirement for pharmacy technician full-time equivalents (FTEs) was reduced to 55.38 from 132.66 and that of FTE pharmacists increased to 117.61 from 46.84 [19]. These gains were achieved without significant increases in staffing. Capilli et al. (2021), when reporting improved productivity and reduced medication turnaround time (TAT), identified that, indeed, the robotic system for chemotherapy preparation does improve workforce efficiency; evidence that there could be a version of economic viability here is proven because of reduced drug wastage and in most cases improved workforce efficiency and reduced operational cost [20]. Hence, their evidence consistently supports the financial viability of PAS implementation, with benefits accrued through reduced drug waste, improved workforce efficiency, and reduced operational costs (Table 2).

**Table 2:** Studies evaluating economic evaluation and cost-effectiveness of PAS PAS: pharmacy automation system, ADS: automated dispensing system, ICU: intensive care unit, ADM: automated dispensing machine, FTE: full-time equivalent, MTAT: medication turnaround time

Author	Study design	Population	Intervention	Comparison	Outcomes	Results
Baan et al. (2022) [16]	Descriptive study	Hospital pharmacy	Vial-sharing of expensive drugs in automated compounding	No vial-sharing (standard practice)	Cost savings, drug wastage reduction	Significant cost savings and reduction in drug wastage due to vial-sharing strategy
Chapuis et al. (2015) [18]	Financial analysis	Three surgical ICUs in a university hospital	Implementation of ADS	Traditional floor stock system	Economic impact, time savings, cost reductions	Reduced nurse time on medication-related activities, reduced drug storage and expired drug costs, positive net present value, significant financial savings
Noparatayaporn et al. (2017) [19]	Comparison study	2,100-bed university hospital	ADM system	Manual dispensing system	Human resource requirement FTEs	The manual system required 46.84 FTEs of pharmacists and 132.66 FTEs of pharmacy technicians; the ADM system required 117.61 FTEs of pharmacists and 55.38 FTEs of pharmacy technicians; the modified ADM system required 69.78 FTEs of pharmacists and 51.90 FTEs of pharmacy technicians​​
Capilli et al. (2021) [20]	Comparative observational study	Pharmacy department in a large oncology hospital	Implementation of robotic systems for chemotherapy preparation	Manual preparation of chemotherapy	Productivity, workload MTAT	Increased productivity and efficiency, reduced MTAT, no significant increase in staff required
Liu et al. (2022) [17]	Economic evaluation	Hospitals implementing vial-sharing strategies	Vial-sharing for expensive drugs	Standard single-use vial practice	Cost savings, drug wastage reduction	Significant cost savings and reduction in drug wastage through vial-sharing strategy

Error Reduction and Safety Improvement

Error reduction and safety improvements will benefit care delivery in several settings, as shown by studies conducted between 2012 and 2022. Vicente Oliveros et al. (2017) reported that medication administration recording errors were reduced from 48.0% to 36.9% after implementing electronic medication administration records (eMAR) [21]. Epstein et al. (2016) found that ADC improves reconciliation accuracy and efficiency for controlled substances [22]. Seger et al. (2012) [23] and Werumeus Buning et al. (2020) [24] improved medication accuracy and staff safety during antineoplastic preparation, respectively, with the latter noting that environmental contamination was lower with the APOTECAchemo robotic system. Alanazi et al. (2022) [25] and ML et al. (2021) [26] reported reduced medication error rates and improved patient safety at tertiary care hospitals with automated drug-dispensing systems. Nanni et al. (2020) reported a 100% elimination of expired medications through regular ADC audits [27]. In contrast, Pelayo et al. (2013) found mixed results with semiautomated unit dose systems, one indicating reduced preparation time but increased administration time [28]. PAS implementation generally enhances medication safety and reduces errors, although with varying successes in specific applications and only when proper implementation protocols are followed (Table 3).

**Table 3:** Studies evaluating error reduction and safety improvement with the use of PAS PAS: pharmacy automation system, ADC: automated dispensing cabinet, eMAR: electronic medication administration record, ME-MAR: medication error-medication administration record, ADS: automated dispensing system, ADDs: automated drug-dispensing systems, TDDs: traditional drug-dispensing systems, MEs: medication errors, UDS: unit dose system

Author	Study design	Population	Intervention	Comparison	Outcomes	Results
Epstein et al. (2016) [22]	Controlled substance reconciliation study	Hospital pharmacy and nursing staff	Near real-time drug transactions captured from ADC	Traditional manual drug transaction reconciliation	Reconciliation accuracy, time efficiency	Improved accuracy in controlled substance reconciliation, reduced reconciliation time
Vicente Oliveros et al. (2017) [21]	Before-and-after quasi-experimental study	947-bed teaching hospital	Implementation of eMAR application	Traditional paper-based medication administration records	ME-MAR, patient safety	The ME-MAR rate decreased from 48.0% to 36.9%; significant reduction in errors and potential risks with eMAR implementation
ML et al. (2021) [26]	Financial analysis	ICU patients at the University Hospital of Grenoble, France	ADS	Traditional manual medication distribution systems	Economic impact and efficiency	ADS implementation reduced MEs, improved inventory management, and significant cost savings
Alanazi et al. (2022) [25]	Observational study	Two governmental tertiary care hospitals in Saudi Arabia	ADDs	TDDs	Patient safety, medication error rate	Improved patient safety and reduced medication error rates with ADDs
Seger et al. (2012) [23]	Direct observation trial	Academic medical center pharmacy staff	Robotic antineoplastic and adjuvant medication compounding	Manual antineoplastic and adjuvant drug preparation	Serious MEs, staff safety events, medication accuracy, medication preparation time, costs of ancillary materials, and personnel time	Serious MEs: no change, staff safety events: reduced, medication accuracy: improved, ancillary material costs: decreased
Nanni et al. (2020) [27]	Pre-post analysis	Four ADCs at an academic medical center	Monthly audits of ADC pockets	Traditional methods to identify expired medications	Rates of finding expired medications	ADC with full audits had no expired doses; overall improvement in expired-dose rates for all audited ADCs
Werumeus Buning et al. (2020) [24]	Comparative observational study	Pharmacy of the OLVG Hospital, Amsterdam	Robotic system (APOTECAchemo)	Manual compounding	Environmental contamination, external contamination, external cross-contamination	Lower external cross-contamination and improved environmental contamination after cleaning optimization with the robotic system
Pelayo et al. (2013) [28]	Safety-oriented usability test	Nine nurses with an average of 5.5 years of clinical experience	Implement a semiautomated UDS designed to assist in preparing and administering oral route drugs	Manual preparation procedures	Task completion time, error rates, user satisfaction, and number of drugs correctly recalled	The UDS reduced preparation time but increased administration time

Efficiency and Workflow Improvement

An analysis of studies from 2014 to 2024 that assessed efficiency and workflow using the PAS showed that it was effective in most settings. The ROWA Vmax robotic system reduced the dispensing error rates (1.31%-0.63%) and stock-out ratios (0.85%-0.17%) to very low values. The new technology from the PharmaHelp system achieved a more than threefold increase in weight accuracy and a twofold reduction in processing time for aseptic preparation, as recorded by Schoening et al. (2015) [29] and Goyanes et al. (2019) [30], respectively. The CytoCare robot achieved improved medication accuracy when the associated costs were higher; this was reflected in another case where it demonstrated less adaptability than expected. Sandler Topelius et al. (2024) produced more accurate results than MiniLab Printers [31]. An early evaluation from the study by DoseEdge showed an improvement in error detection and documentation accuracy, which also corresponded to the findings of Moniz et al. (2014) [32]. According to Ramachandram et al. (2023), automated tablet dispensing and packaging systems (ATDPS) significantly reduce employees' workloads and increase job satisfaction [33]. Berdot et al. (2018) found automated drug-dispensing cabinets to be expensive initially, but they had long-term benefits related to time for preparation and reduced errors [34]. Denman et al. (2015) [35] and Tamblyn et al. (2017) [36] documented improved accuracy and efficiency in automated systems compared to manual processes (Table 4).

**Table 4:** Studies evaluating efficiency and workflow management with the use of PAS PAS: pharmacy automation system, ANSC: automated non-sterile compounding, ATDPS: automated tablet dispensing and packaging system, ADCs: automated dispensing cabinets, TFSS: traditional floor stock systems, MEs: medication errors

Author	Study design	Population	Intervention	Comparison	Outcomes	Results
Sandler Topelius et al. (2024) [31]	Comparative analysis	Various tablet formulations and sizes	Pharma Printer	MiniLab Printers	Accuracy, consistency, efficiency	Superior accuracy and content uniformity with the Pharma Printer across multiple tablet sizes
Goyanes et al. (2019) [30]	Observational study	Pharmacy department at Brigham and Women's/Dana-Farber Cancer Care Center	Robotic antineoplastic preparation system (CytoCare robot)	Manual preparation of antineoplastics and adjuvant medications	Patient safety, staff safety, workflow efficiencies, medication accuracy, costs	Reduced medication preparation errors, decreased staff safety events, improved accuracy in drug preparation, higher costs associated with robotic preparation compared to manual methods
Denman et al. (2015) [35]	Comparative study	Leeds Teaching Hospitals NHS Trust	Automated upload process	Manual paper-based system	Data accuracy, administrative time	Improved accuracy, reduced time
Tamblyn et al. (2017) [36]	Observational study	Clinical pharmacy services at the University of Arizona	ANSC	Manual compounding	Consistency, efficiency, cost-effectiveness	Improved consistency and efficiency, cost savings with ANSC
Rodriguez-Gonzalez et al. (2018) [37]	Prospective before-and-after medication error study	Outpatient hospital pharmacy in a 1,300-bed tertiary teaching hospital in Madrid, Spain	Robotic original pack dispensing system (ROWA Vmax)	Manual dispensing using a barcode-controlled system	Medication dispensing errors, stock management quality, staff satisfaction	The dispensing error rate was reduced from 1.31% to 0.63%, the stock-out ratio was decreased from 0.85% to 0.17%, and staff satisfaction improved, especially among pharmacists
Schoening et al. (2015) [29]	Prospective before-and-after study	Hospital pharmacy, including the National Cancer Center	PharmaHelp semiautomated aseptic preparation system	Manual volumetric-based preparation	Weight conformity, microbiological contamination, productivity	Improved weight accuracy, significantly reduced manual preprocessing and postprocessing times, and enhanced staff safety
Moniz et al. (2014) [32]	Observational study	Pharmacy department at Boston Children's Hospital	IV compounding workflow management system (DoseEdge)	Traditional paper-based documentation system	Compounding errors, documentation accuracy, preparation time	Improved detection of preparation errors and documentation errors
Ramachandram et al. (2023) [33]	Observational, cross-sectional survey	21 pharmacists and 18 pharmacy assistants	ATDPS	Traditional manual dispensing and packaging	Workload, task duration, job satisfaction	Reduced workload, improved job satisfaction, perceived benefits for patient safety, and decreased MEs
Berdot et al. (2018) [34]	Quasi-experimental multicenter study	Two French hospitals (all wards of the two hospitals were included in the study)	Implementation and use of ADCs	TFSS	Compared costs, drug management efficiency, nurse satisfaction, and medication process errors	Significant initial investment cost was offset by long-term gains in preparation time and reduced medication process errors

Quantitative Analysis

Nine of the 32 studies were selected for quantitative analysis based on comprehensive quantitative data suitable for effect size calculation. These studies reported detailed statistical information, including sample sizes, outcome measures with numerical data, and measures of variability (such as standard deviations or confidence intervals). This level of detail was essential for calculating the standardized effect sizes and conducting a meta-analysis.

An analysis of nine studies (2012-2022) assessing PAS suggested positive outcomes across diverse healthcare settings. The effect sizes indicated marked improvements in medication safety and efficiency. Chapuis et al. (2015) reported the most significant positive effect size for ADC in the ICU (d=11.22) [18]. Compared with traditional systems, ADC also showed substantial benefits (d=8.97). The automated system showed varying effects on reducing errors across studies. Notably, the study by Alanazi et al. (2022) reported a large negative effect size (-3.16), indicating that in their specific context, the automated drug-dispensing system (ADDs) was associated with significantly higher error rates compared to the traditional drug-dispensing system (TDDs) [25]. Robotic implementations were related to moderate effects; Rodriguez-Gonzalez et al. (2017) found an effect size of 0.69 for error reduction [37]. Vicente Oliveros et al. (2016) noted that eMAR was associated with lower effects but positive ones: 0.23 [21]. According to Werumeus Buning et al. (2019), robotic systems reduced contamination rates (odds ratio (OR)=0.53) [24]. The data prove that while all PAS did improve, the benefit size and setting differed for each type of automation, with a more significant effect seen in comprehensive system implementations (Table 5).

**Table 5:** Characteristics of the included studies This table presents the characteristics of the studies included in the quantitative analysis. It consists of the sample size, intervention, comparison, outcome measures, and effect sizes (d or OR) for each study. ADDs: automated drug-dispensing systems, TDDs: traditional drug-dispensing systems, ADCs: automated dispensing cabinets, TFSS: traditional floor stock systems, ADS: automated dispensing system, eMAR: electronic medication administration record, MAR: medication administration record, OR: odds ratio

Study	Sample size	Intervention	Comparison	Outcome measures	Effect size (d or OR)
Alanazi et al. [25]	40	ADDs	TDDs	Error rates, efficiency, inventory management, patient safety	-3.16
Berdot et al. [34]	2 hospitals	ADCs	TFSS	Cost, error rates, nurse satisfaction	8.97
Chapuis et al. [18]	3 ICUs	ADS	Floor stock	Cost, time savings, error rates	11.22
Epstein et al. [22]	1,421/1,483	ADCs	Manual reconciliation	Error rates, workflow efficiency	3.58 (OR)
Rodriguez-Gonzalez et al. [37]	3,284/3,004	Robotic system	Manual system	Error rates, stock management, staff satisfaction	0.69
Seger et al. [23]	1,421/972	Robotic preparation	Manual preparation	Error rates, staff safety, costs	0.88 (OR)
Vicente Oliveros et al. [21]	2,835/2,621	eMAR	Manual MAR	Error rates	0.23
Werumeus Buning et al. [24]	113/171	Robotic system	Manual system	Contamination rates	0.53 (OR)
Yaniv and Knoer et al. [14]	7,384	Robotic compounding	Manual compounding	Error rates, costs	-1

Analysis of the effect sizes revealed substantial variation in the impact of PAS. The most significant positive effects were observed by Berdot et al. (2015) [34] and Chapuis et al. (2015) [18], showing effect sizes of 8.97 (CI: -30981.354 to 30999.294) and 11.22 (CI: -7736.366 to 7758.806), respectively. However, their wide confidence intervals suggest considerable uncertainty. Rodriguez-Gonzalez et al. (2017) demonstrated the most precise results with an effect size of 0.69 (CI: 0.671 to 0.709) and the highest statistical weight (10412.331), indicating strong reliability [37]. Epstein et al. (2016) showed moderate positive effects (1.275, CI: 0.719 to 1.832) [22], while Alanazi et al. (2022) reported significant adverse effects (-3.16, CI: -4.708 to -1.612) [25]. Yaniv and Knoer (2012) also showed negative effects (-1, CI: -1.055 to -0.945) but with higher precision [14]. The remaining studies by Seger et al. (2012) [23], Vicente Oliveros et al. (2016) [21], and Werumeus Buning et al. (2019) [24] showed modest effects ranging from -0.635 to 0.23, with overlapping confidence intervals suggesting similar underlying impacts. A random-effects model was used for quantitative analysis to account for study variability (Table 6). The overall mean effect size was 0.505 (95% CI: 0.487 to 0.523), indicating a moderately positive effect of PAS compared to traditional methods. Substantial heterogeneity was observed among the studies (I² = 99.76%).

**Table 6:** Effect sizes and confidence intervals for individual studies This table shows the effect sizes and confidence intervals for the individual studies included in the quantitative analysis. It provides detailed statistical information, including each study's effect size, variance, standard error, weight, and confidence intervals. CI: confidence interval

Study	Effect size	Variance	Standard error	Weight	CI (lower)	CI (upper)
Alanazi et al. [25]	-3.16	0.624	0.79	1.602	-4.708	-1.612
Berdot et al. [34]	8.97	250000000	15811.39	0.000000004	-30981.354	30999.294
Chapuis et al. [18]	11.22	15625000	3952.85	0.000000064	-7736.366	7758.806
Epstein et al. [22]	1.275	0.08	0.284	12.394	0.719	1.832
Rodriguez-Gonzalez et al. [37]	0.69	0.000096	0.01	10412.331	0.671	0.709
Seger et al. [23]	-0.128	0.254	0.504	3.938	-1.116	0.86
Vicente Oliveros et al. [21]	0.23	0.226	0.475	4.432	-0.701	1.161
Werumeus Buning et al. [24]	-0.635	0.153	0.391	6.545	-1.401	0.131
Yaniv and Knoer et al. [14]	-1	0.000784	0.028	1275.51	-1.055	-0.945

Risk of Bias in the Included Studies

**Figure 2:**
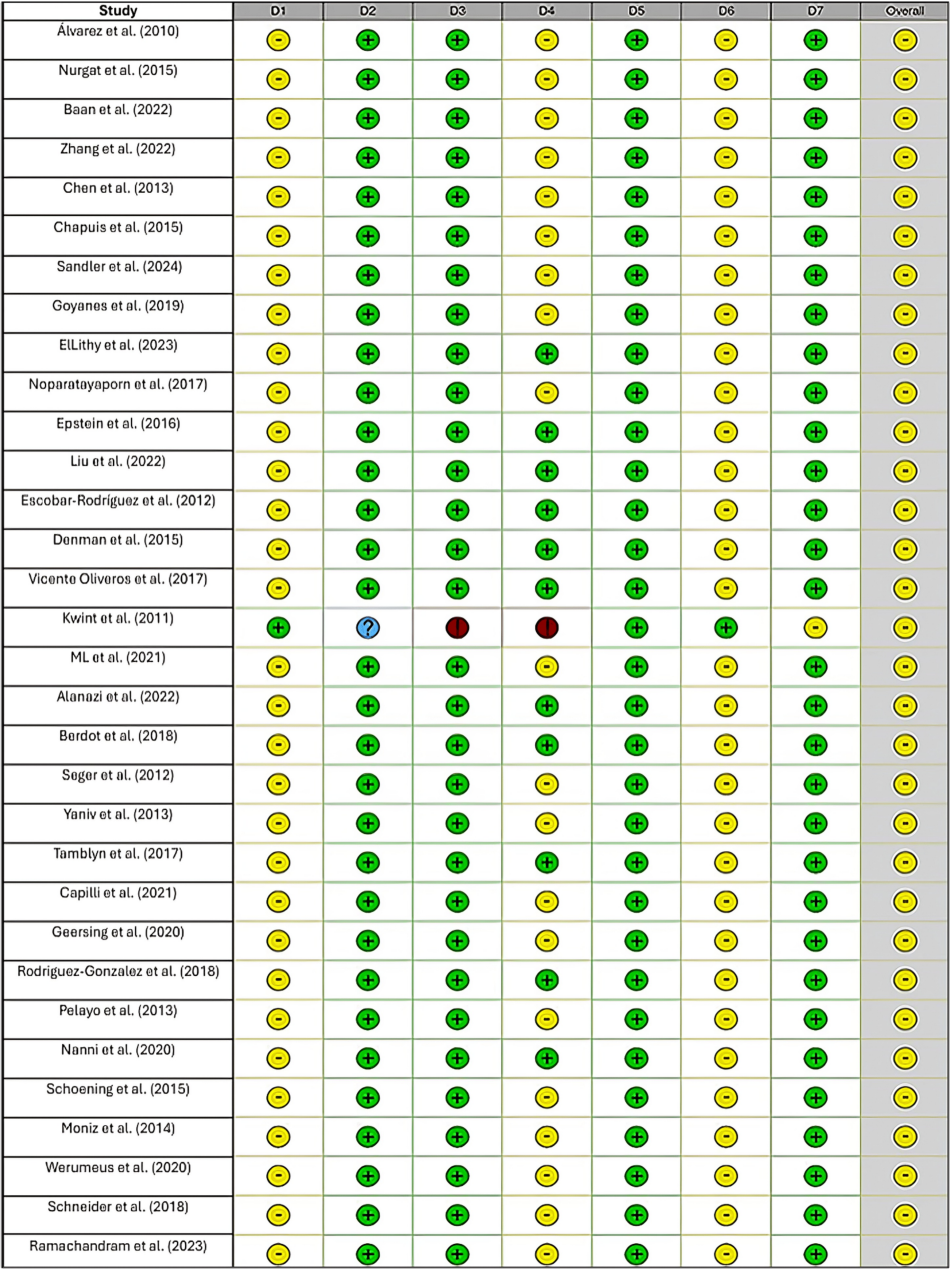
Traffic light plot showing risk of bias in individual studies This figure visually represents the risk of bias assessment for each included study across the different domains. Studies shown include the following: Álvarez Díaz et al. (2010) [6], Nurgat et al. (2015) [8], Chen et al. (2013) [10], Zhang et al. (2022) [7], Dinesh et al. (2015) [35], ElLithy et al. (2023) [11], Escobar-Rodriguez et al. (2012) [12], Schneider (2018) [13], Chapuis et al. (2015) [18], Berdot et al. (2018) [34], Vicente Oliveros et al. (2017) [21], ML et al. (2021) [26], Alanazi et al. (2022) [25], Rodriguez-Gonzalez et al. (2018) [37], Moniz et al. (2014) [32], Werumeus Buning et al. (2020) [24], Pelayo et al. (2013) [28], Schoening et al. (2015) [29], Capilli et al. (2021) [20], Geersing et al. (2020) [15], Yaniv and Knoer (2013) [14], Tamblyn et al. (2017) [36], Ramachandram et al. (2023) [33], Goyanes et al. (2019) [30], and Sandler Topelius et al. (2024) [31]. Green circles indicate low risk, yellow circles indicate moderate risk, red circles indicate high risk, and blue circle question marks indicate missing information. The assessment covers seven domains (D1-D7): confounding, participant selection, intervention classification, deviation from interventions, missing data, outcome measurement, and result reporting bias.

The overall risk of bias across the studies was moderate, with a significant portion showing low risk and a small number indicating critical risk (Figure 2). These visual tools suggest that while many studies included in the review are of moderate quality with some low-risk assessments, specific areas, particularly confounding, deviations from intended interventions, and measurement of outcomes, require careful consideration when interpreting the findings.

Discussion

This systematic review assessed the effects and efficiency of PAS compared with TPS in hospitals. The main measures used to evaluate the effectiveness of the implemented interventions were rates of MEs, improvement in efficiency, cost-effectiveness, and patient status results.

Medication Errors

Based on these results, it can be postulated that PAS use is associated with marked reductions in MEs. The most significant impact was observed with the use of technology with ADS and CPOE systems. Similar to our findings, the study conducted by Kimble and Chandra (2001) revealed that potential MEs decreased after implementing PAS [38]. A similar study by Olubunmi Afolabi and Oyedepo Oyebisi (2007) found that automation positively affected decreasing MEs [39]. However, to the best extent, required training and technical support must be provided to achieve these results.

Improvement in Efficiency

Automated systems enhance the effectiveness of pharmacy operations, particularly dispensing medication and tracking stock. This also resulted in shorter turnaround times and helped pharmacists devote much of their time to clinical activities, thus improving the working cycle. Zhou et al. (2015) asserted that integrating PAS increased efficiency and minimized space consumption and workload, which connotes our results in enhancing the workflow [40]. Similarly, Shou and Jin (2012) postulated that internal dispensing errors were reduced and work efficiency increased by adopting an automated outpatient pharmacy system [41].

Cost-Effectiveness

Although PAS in healthcare facilities are expensive to implement, long-term operating costs are lower because of reduced labor costs and decreased medication waste. Sng et al. (2018) conducted an economic evaluation for ADS, which supported increased productivity and reduced costs in outpatient and community settings, although they noted that staff satisfaction remained unchanged [42].

Patient Outcomes

Optimization of drug distribution and effectiveness of medications contributed to enhanced patient satisfaction and lower rates of adverse drug events. In their study, Al-Mofleh et al. (2023) noted improved patient safety and satisfaction with a comprehensive PAS, which is consistent with our results [43]. Batson et al. (2020) highlighted the positive outcomes of PAS, reduced MEs, and improved cost-effectiveness [1]. These comparisons strengthen the argument of this review, showing that the vast literature supports PAS and contributes positively to the reduction of MEs, increased efficiency, cost control, and positive patient outcomes. However, adoption and involvement persist and demand proper training and technical support.

Implications for Practice

PAS has been regarded as highly efficient and has decreased clinical costs and, more importantly, drug malpractice, thereby enhancing patient treatment. Automation can streamline pharmacy operations, allowing pharmacists to allocate more time to patient-centered clinical activities. In addition, using PAS is justified because the benefits of these systems are far greater than the initial funding costs.

Strengths, Limitations, and Future Research

We used an extensive search strategy to evaluate relevant papers in the research databases. Rigorous screening and selection processes were performed to reduce the risk of bias. However, significant limitations include heterogeneity among studies regarding the setting, sample size, and type of outcome assessed, all of which can affect the generalizability of findings. Therefore, although results support the benefits of PAS, more extensive research is needed to address the existing gaps in the literature. Comprehensive cost-benefit analyses are required to determine the profitability of each automation in several aspects of a hospital. Moreover, more efforts should be made to assess the impact of automation on user satisfaction among pharmacists and pharmacy technicians to understand the human factors that influence successful implementation.

## Conclusions

Compared with TPS, PAS can mitigate MEs, enhance operational efficiency, and favorably influence patient outcomes. Despite substantial initial capital expenditure in most instances, the subsequent costs and benefits associated with a hospital's operational performance justify the adoption of these technologies. However, while PAS has the potential to improve various aspects of hospital pharmacy operations, its successful implementation necessitates careful consideration of context-specific factors, adequate staff training, and continuous system monitoring. As technology advances, ongoing research and evaluation will be essential to ensure that PAS is optimally utilized to enhance medication safety and overall healthcare quality.
